# Dynamics of the Full Length and Mutated Heat Shock Factor 1 in Human Cells

**DOI:** 10.1371/journal.pone.0067566

**Published:** 2013-07-08

**Authors:** Gaëtan Herbomel, Meike Kloster-Landsberg, Eric G. Folco, Edwige Col, Yves Usson, Claire Vourc’h, Antoine Delon, Catherine Souchier

**Affiliations:** 1 INSERM, University Grenoble 1, IAB CRI U823 team 10, La Tronche, France; 2 University Grenoble 1, CNRS, LIPhy UMR 5588, St Martin d’Hères, France; 3 University Grenoble I, CNRS, TIMC-IMAG UMR5525, La Tronche, France; University of South Florida, United States of America

## Abstract

Heat shock factor 1 is the key transcription factor of the heat shock response. Its function is to protect the cell against the deleterious effects of stress. Upon stress, HSF1 binds to and transcribes hsp genes and repeated satellite III (sat III) sequences present at the 9q12 locus. HSF1 binding to pericentric sat III sequences forms structures known as nuclear stress bodies (nSBs). nSBs represent a natural amplification of RNA pol II dependent transcription sites. Dynamics of HSF1 and of deletion mutants were studied in living cells using multi-confocal Fluorescence Correlation Spectroscopy (mFCS) and Fluorescence Recovery After Photobleaching (FRAP). In this paper, we show that HSF1 dynamics modifications upon heat shock result from both formation of high molecular weight complexes and increased HSF1 interactions with chromatin. These interactions involve both DNA binding with Heat Shock Element (HSE) and sat III sequences and a more transient sequence-independent binding likely corresponding to a search for more specific targets. We find that the trimerization domain is required for low affinity interactions with chromatin while the DNA binding domain is required for site-specific interactions of HSF1 with DNA.

## Introduction

Recent advances in microscopy and in fluorescent protein tags [1], [2] make it possible to characterize molecular dynamics in living cells. Mostly based on Fluorescence Recovery After Photobleaching (FRAP) data, active transcription factors are known to diffuse rapidly into the nucleoplasm and to display “hit and run” interactions with their targets [3], [4], [5], [6]. Therefore, the general behavior of transcription factor kinetics can be described and fitted by diffusion-reaction models [7], [8], [9].

Studies of transcription factors show that their dynamics are slowed down upon activation, to an extent depending on the transcription factor and biological model considered (endogenous versus artificial gene array [10]). For example, the fluorescence half-recovery time of the estrogen nuclear receptor in the nucleoplasm increases from 1 s to 5 s when 17β-estradiol is added and to 12 s when measurements are performed on progesterone responsive gene-array [11]. In this general context, the dynamics of HSF1 on heat shock genes in a model of Drosophila polytenic chromosomes appears to be significantly slower (t_1/2_ ≈ 6 min) [12], while, in contrast, we recently showed that HSF1 is more dynamics in the nucleoplasm of human U87 cells [13] than in polytenic chromosomes.

HSF1 isoform is the key transcription factor of the heat shock response in vertebrates [14], [15]. It is composed of four main domains, namely DNA binding, trimerization, regulatory and trans-activation domains [14], [15]. Upon heat shock, HSF1 undergoes trimerization and post-translational modifications. Activated HSF1 binds to HSEs present in the promoter of heat shock genes. Moreover, in human cells, HSF1 relocates within nuclear Stress Bodies (nSBs) [16]. NSBs form primarily at the pericentromeric region of human chromosome 9 (9q12) through direct binding of HSF1 with satellite III (sat III) repeated sequences. HSF1 interaction with sat III sequences involves its DNA binding domain and represents a prerequisite for the RNA-pol II dependent transcription of sat III sequences [17]. The presence of nSBs in human cells makes it possible to follow the dynamics of HSF1, by in situ approaches, at endogenous specific targets [18].

Fluorescence Correlation Spectroscopy (FCS) is a more recent approach complementary to FRAP. It is a sensitive non-destructive technique, well adapted to low concentrations of fluorescent molecules (<10 µM) and to rapid dynamics (<1 s) [19], [20], [21]. In this paper, our objective is a better understanding of HSF1 dynamics involving rapid and slow processes, in unstressed and stressed living cells, by combining multiconfocal FCS (mFCS) and FRAP approaches. In addition, we took advantage of nSBs to study HSF1 dynamics at specific HSF1-DNA binding sites. Using HSF1 mutants, we have also examined the role of different functional domains of HSF1. The size of HSF1- containing complexes and the percentage of bound HSF1 fractions deduced from mFCS and FRAP data were also compared to those obtained from glycerol fractionation and salt extraction experiments performed in living cells.

## Materials and Methods

### Plasmid Constructs

The coding sequence for human HSF1 was obtained after PCR amplification and cloned into a peGFP N3 vector (Clontech Laboratories Mountain View, CA) or into a pcDNA3 TagRFP-T vector (from R. Tsien, [1]). The plasmid expressing the HSF1 K80Q-eGFP mutant was created using the QuikChange II Site-Directed Mutagenesis Kits (Agilent Technologies, Santa Clara, CA). The K80Q is a point mutation mimicking acetylation and disrupting DNA binding activity [22]. Plasmids expressing HSF1 ΔTRIM-eGFP and HSF1 ΔDBD-eGFP were obtained by an overlap PCR and insertion into the peGFP N3 vector (Clontech). The plasmids coding for the human wild-type HSF1-eGFP, HSF1-K80Q-eGFP, HSF1 ΔDBD-eGFP, HSF1 ΔTRIM-eGFP, resistant to the siRNAs used for silencing endogenous HSF1 proteins were obtained by mutating two bases (G573A and C576T) using the QuikChange II Site-Directed Mutagenesis Kits (Agilent Technologies, Santa Clara, CA). All plasmids were verified by sequencing (GATC Biotech, Constance, Germany).

### Cell Lines and Transfection

HeLa cells were cultured in Dulbecco’s modified Eagle’s medium (DMEM, PAA, Pashing, Austria) supplemented with 10% (v/v) fetal bovine serum (FBS), 2% L-glutamine (4 mM) and 100 units per ml penicillin and 100 µg/ml streptomycin (Gibco, life Technologies, Carlsbad, CA), and grown in 5% CO_2_ atmosphere at 37°C. Immortalized MEFs from HSF1 null mice [23] were cultured in Hela medium added with 1% non essential amino acid and 50 µM β-mercaptoethanol. Transfections with plasmids expressing the human full-length or mutated HSF1-eGFP were performed with lipofectamine™ 2000 transfection reagent (Life Technologies, Carlsbad, CA). Stable HSF1-eGFP cell lines were established using 600 µg/ml geneticin (Gibco, Life Technologies) and low transfected single cells were selected by flow cell sorting system (FACSAria, Beckton Dickinson, San Jose, CA). Two days before the experiments, 2.5×10^5^ cells were plated per culture dish (Bioptechs, Butler, PA) or one-well chambered coverglass Lab-Tek (Nunc, Roskilde, Dk). Microscopy analysis was performed in DMEM without phenol red, 1% FBS, 2% L-glutamine and added with 10 mM Hepes. HSF1 depletion was performed by two rounds of specific siRNA treatment at 24-hour interval. In the first round, cells were transfected with siRNA (75 nM) using lipofectamine RNAiMAX (Life Technologies, Carlsbad, CA). In the second round, cells were transfected with siRNA (25 nM) and added or not with 1 µg of a plasmid of interest using lipofectamine™ 2000.

### mFCS Acquisition

FCS is based on the measurement of fluorescence fluctuations in a small confocal volume [19], [20], [21]. mFCS was performed on a home built system detailed previously [13]. Briefly, the system makes it possible to perform a simultaneous FCS analysis of five aligned points located within the nucleus. It is based on an inverted microscope stand (Olympus IX70, Tokyo, Japan) equipped with a 60× water objective lens (PlanApochromat, NA = 1.2) and with a temperature control system including stage and objective controls (Delta T, Bioptechs, PA, USA). Its main difference with a commercial FCS system is that several laser spots are generated by a spatial light modulator (SLM) and that the fluorescence detector is a 128×128 pixels Electron Multiplied CCD camera (EMCCD, iXon+DU860, Andor Technology, Belfast, Ireland). Each pixel of the camera acts as an individual pinhole for the parallel multi-spot FCS measurements and, by positioning the spots on the bottom row of the EMCCD camera, the frame acquisition rate reaches 70 kHz. A MATLAB (MathWorks, Natick, MA) interface was developed for performing the mFCS data acquisition and processing. In addition, a CCD camera (Clara, Andor Technology) was used for imaging the cells. Each measurement represents the average value of five successive 10 s acquisitions. Corrections were done for electronic offset, slow fluctuations, fluorescence overlap between close spots, unfocused background and need a supplementary 10 s acquisition with the laser off. Heat shock was performed at 43°C during 1 hour under the microscope. Analyses of MEFs were performed on a FCS system (confocor II, Zeiss, Jena, Germany).

### FRAP Analysis

In the FRAP technique, a small region of interest (ROI) is photobleached by a brief exposure at a high laser intensity and the subsequent fluorescence recovery is monitored at attenuated laser intensity. Confocal images and Fluorescence Recovery After Photobleaching “FRAP” experiments were performed on a confocal laser scanning microscope (CLSM, LSM710, Zeiss, Jena, Germany) equipped with a 40x water immersion objective (C-Apochromat, 1.2 NA). During the experiments, cells were maintained at 37°C in an atmosphere containing 5% C0_2_, using a large incubator system (Zeiss, Germany). Heat shock was performed at 43°C during 1 h using a heating plate close to the microscope. Comparative analysis was done between unstressed and stressed cells, and the same set of nuclei was analyzed in both conditions.

Fluorescence excitation was performed with the 488 nm line of an argon laser combined, for photobleaching, with a 405 nm diode pumped solid state laser “DPSS”. The emission fluorescence intensity was measured through a long pass filter (LP 505 nm) by a photomultiplier (PMT). Fluorescence was briefly photobleached (1 iteration, 80 ms) within a small circular region of interest (ROI) (r = 1.04 µm) and line acquisition (128 pixels corresponding to 13.3 µm) was performed at high rate (3.78 ms) during 12 s or longer when necessary [24].

Fluorescence intensities during recovery were estimated in and outside the photobleaching ROI using MetaMorph image analysis software (Molecular Devices, USA). Data were corrected for background, photobleaching and global nuclear fluorescence loss due to photobleaching, according to equation 1.
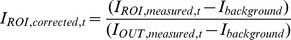
with I representing the fluorescence intensity of the line pixels inside (I_ROI_) or outside (I_OUT_) the photobleaching ROI, and I_background_ the fluorescence intensity measured outside the cells. For smoothing out short-term fluctuations, data of progressive consecutive times were averaged paying attention to the photobleaching pulse duration and to the start of the recovery.

### FCS Modeling

FCS autocorrelation curves were analyzed according to the reaction dominant case of the diffusion interaction models [7]:
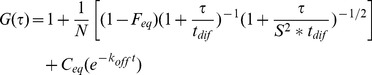
with N the number of molecules, F_eq_, the fraction of free molecules, t_dif_ the diffusion time, S the structural parameter (ratio of the longitudinal to the transverse radii of the confocal volume) and k_off_ the dissociation rate and:



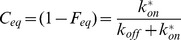
with C_eq_ representing the fraction of bound molecules, 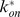 the association rate constant that incorporates the equilibrium concentration of vacant binding sites 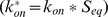. However, an additional temporal component was added due to a superimposed oscillation appearing at very long lag times (≈1 s) in mFCS curves [13]. The adjustment was performed using MATLAB software. For diffusion standardization, the recent value estimated by dual focus FCS [25] for R6G was used to calculate D_R6G_ = 555 µm^2^/s at 37°C and D_R6G_ = 634 µm^2^/s at 43°C.

Diffusion constant was calculated according to:
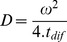
with ω, (0.251 µm), the transverse radius of the confocal volume.

### FRAP Modeling

FRAP was fitted according to the pure diffusion or diffusion interaction model [7]. The non linear adjustment was performed using Origin Pro 8 software (Northampton, MA, USA), according to the following equation :



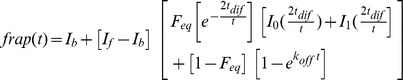
with F_eq_ representing the fraction of free molecules, I_n_ the modified Bessel functions of order n, t_dif_ the diffusion time, I_b_ the relative intensity of bound species just after the photobleaching and I_f_ that at infinite time, k_off_ the dissociation rate constant.

The diffusion constant calculation was calculated from the measured value t_dif_, by taking into account the effect of diffusion during photobleaching according to Kang [26]. To do so, the normalized post-bleach profile was fitted according to the following equation:



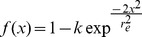
with r_e,_ the effective radius of a post-bleach profile.

The diffusion constant was then estimated according to the following equation:



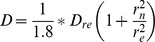
with r_n_ the photobleached ROI: (r_n_ = 1.04 µm).

### Statistical Analysis

mFCS and FRAP data were analyzed using STATA (College Station, Texas).

### Immunolabelling

Cells were fixed in 4% paraformaldehyde in PBS for 5 minutes. After a one-hour incubation in 10% fetal calf serum/0.3% Triton X100/PBS to block non-specific binding sites, cells were incubated for one hour at 37°C with the rabbit anti-HSF1 antibody (1/300, Stressgen, Assay Designs Inc, USA). The antibodies were detected using a goat anti-rabbit antibody coupled to Dylight 549 (KPL, Marylan, USA).

### Western Blot

After extraction with 8 M urea, proteins were analyzed by SDS-PAGE (8%) using specific primary antibodies against HSF1 (ADI-SPA 901, Enzo Life Sciences, Farmingdale, NY) or against α-tubulin (T5168, Sigma-Aldrich, St. Louis, MO).

### Quantitative PCR

RNA was extracted with the Nucleospin RNA II kit (Macherey Nagel, Düren, Germany). For quantitative PCR analysis, first, 1 µg of RNA was retro-transcribed using the transcription first strand cDNA synthesis kit (Roche, Basel, Switzerland). Second, quantitative SYBR-green based PCR assays (Light cycler 480 SYBR green I master, Roche) were performed on 5 ng cDNA for HSP70 amplification or GAPDH used as control or 125 ng for sat III.

### Protein Extraction in Living Cells

Cells were cultured in eight well Lab-Tek chambered coverglasses and subjected to a one-hour heat shock. The protocol of protein extraction was adapted from Sheval, et al. [27]. First, cells were permeabilized for 10 min at 4°C, in a buffer containing 50 mM Tris-HCl (pH 7.6), 5 mM MgCl_2_, 1 mM CuSO_4_, 1 mM PMSF and 0.5% Triton X-100. Cells were then washed with the same buffer without Triton. Protein extraction was performed for 10 min at 4°C in a buffer containing 10 mM EDTA, 20 mM Tris-HCl and various concentrations of NaCl (from 0 to 1 M). Cells were then washed, fixed with 4% PFA and imaged.

### Oligonucleotide Pull-down

Protein extracts from transfected cells were incubated during two hours at 4°C with annealed biotinylated oligonucleotides containing HSE motifs (5′-biotin - AAC-GAG-AAT-CTT-CGA-GAA-TGG-CT-3′ and 5′- AGC-CAT-TCT-CGA-AGA-TTC-TCG-TT-3′) or with annealed scrambled control oligonucleotides (5′-biotin -AAC-GAC-GGT-CGC-TCC-GCC-TGG-CT-3′ and 5′- AGC-CAG-GCG-GAG-CGA-CCG-TCG-TT-3′) (Eurogenetec, Germany). Proteins-biotinlylated oligonucleotide complexes were isolated by incubating samples with streptavidin sepharose high performance beads (Ge Healthcare, UK). DNA-bound proteins were eluted and proteins separated on 8% SDS poly-acrylamide gel electrophoresis were then identified by western blotting using specific antibodies against HSF1 (ADI-SPA 901, Enzo Life Sciences, Farmingdale, NY).

### Fractionation on Glycerol Gradient

Gradients were made in Beckman centrifuge tubes (11×34 mm) by layering three different solutions with glycerol concentrations of 20, 30 and 40% in 20 mM Hepes pH 7.9, 100 mM NaCl, 2.5 mM MgCl_2_, 1 mM DTT, protease inhibitor (complete ULTRA tablet, Roche, Basel, Switzerland), phosphatase inhibitor (PhosSTOP, Roche, Basel, Switzerland) and 100 ng/ml TSA. Gradient was then frozen at -80°C, and kept at 4°C prior ultracentrifugation. Proteins were extracted using LSDB 500 (25 mM Hepes pH 8, 500 mM KCl, 1 mM EDTA, 5 mM MgCl_2_, 10% glycerol, 0.1% NP40, Complete ULTRA, PhosSTOP, 1 mM DTT) for one hour, centrifuged for 25 min and then loaded on the top of the gradients. Ultracentrifugation was made on a Sorval RC M120ex using a RP55s-164 rotor at 53000 rpm during 5 h at 4°C. Fractions were then collected and analyzed by Western Blot.

## Results

**Figure 1:**
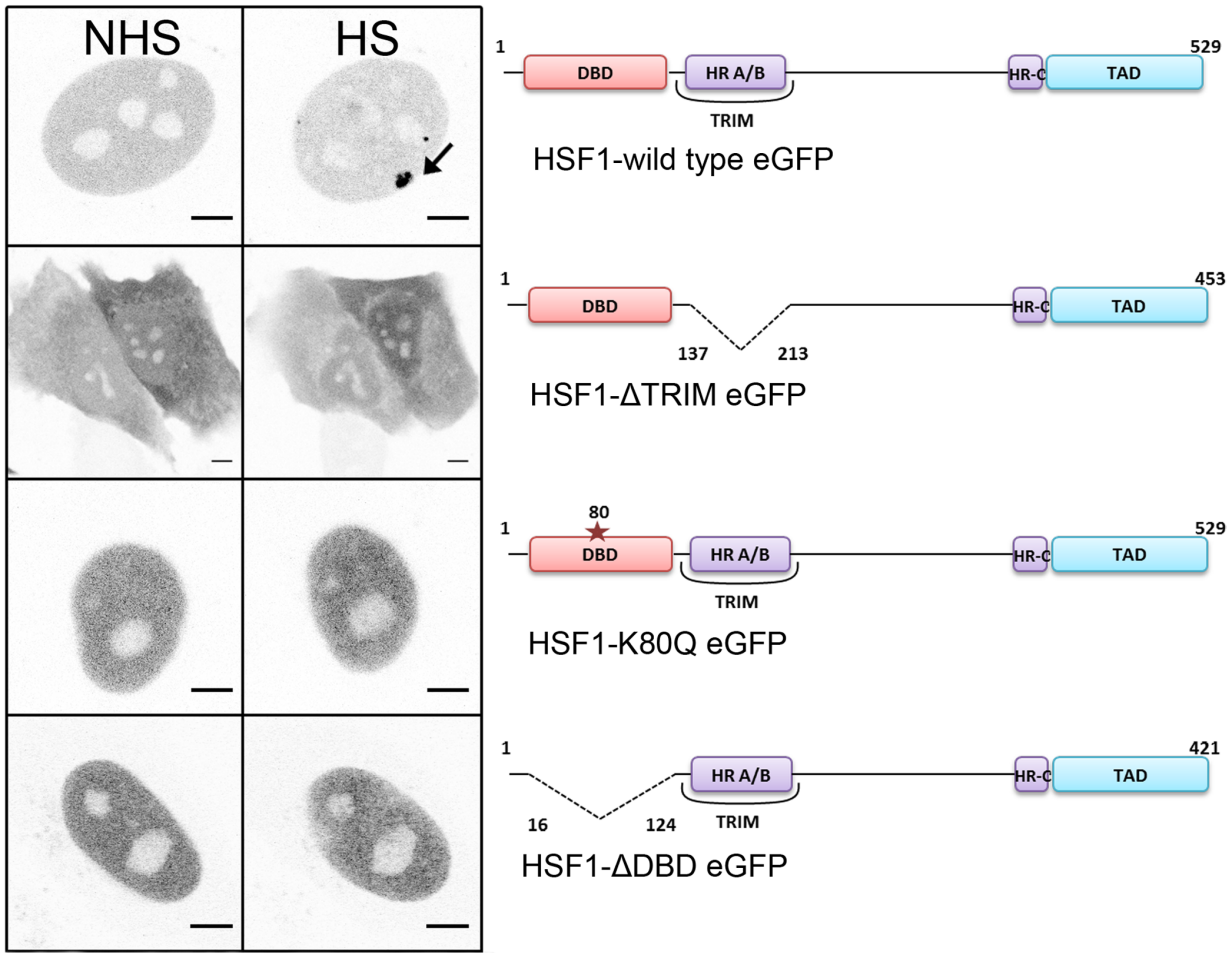
Intracellular localization of HSF1 transcription factor in HeLa cells. CLSM images of wild type HSF1-eGFP, HSF1-ΔTRIM-eGFP, HSF1-K80Q-eGFP and HSF1-ΔDBD-eGFP before (NHS) and after a one hour heat shock (HS). HSF1 is predominantly nuclear except when the trimerization domain is deleted. After a one-hour heat shock, nuclear stress bodies (nSBs, black arrows) are only formed with HSF1-eGFP full length. Scale bar = 5 µm.

The dynamics of full length human HSF1 (WT-HSF1) in living cells, was compared to that of human HSF1 mutated in its DNA binding domain (DBD) (ΔDBD and K80Q) or in its trimerization domain (ΔTRIM) (see Figure 1).

### HSF1-eGFP Localization and Expression Level

**Figure 2:**
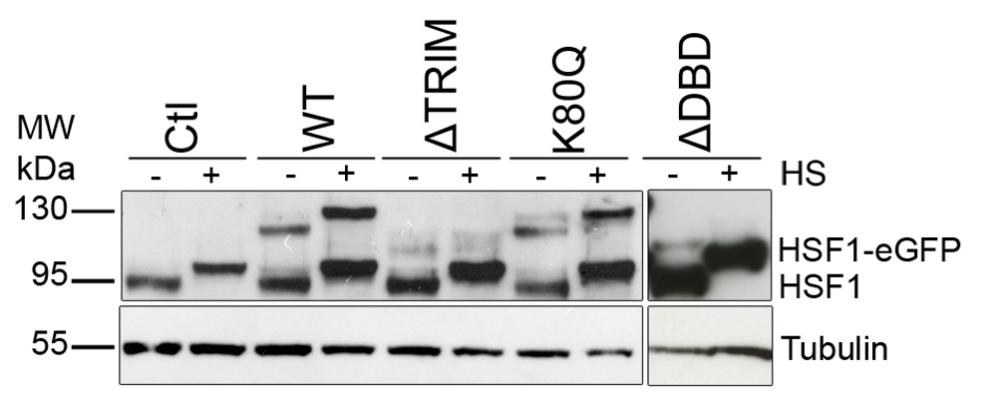
Expression levels of HSF1 and HSF1-eGFP in stable HeLa cell lines. HSF1 western blotting of full protein extracts before (−) and after one hour heat shock (+), in control cells (Ctl), cell lines expressing the full length HSF1-eGFP (wt), the HSF1-eGFP mutant deleted of the trimerization domain (ΔTRIM), the HSF1-eGFP with the punctual mutation (K80Q) and the HSF1-eGFP mutant deleted for the DNA binding domain (ΔDBD). The shifted bands detected after HS (+) represent the phosphorylated form of HSF1.

Full-length HSF1 (WT) and HSF1 mutants fused to the Green Fluorescent Protein (eGFP) were used to establish stable HeLa cell lines. The electrophoretic behavior and the level of expression of each fusion protein were examined by western blot. As shown in Figure 2, WT-HSF1 and mutated HSF1 fusion proteins were all expressed at a level equal or lower to that of endogenous HSF1. GFP-fusion proteins were expressed at the expected size in unstressed cells. In addition, all fusion proteins displayed a delayed migration after heat shock, due to the existence of well-characterized heat-induced phosphorylations [28], [29].

Cells transfected with the different fusion proteins were also analyzed by confocal laser scanning microscopy (CLSM) (Figure 1). As expected, full-length HSF1-eGFP protein was mainly nuclear and relocated into nSBs. No difference between a C*-* or N*-*terminal eGFP fusion was observed (data not shown). Similar to earlier observations [22], [30], both trimerization and DNA binding domains were required for the formation of nSBs, upon heat shock.

### Stress Induced Slow-down of WT-HSF1 and HSF1 Mutated in the DNA Binding Domain

**Figure 3:**
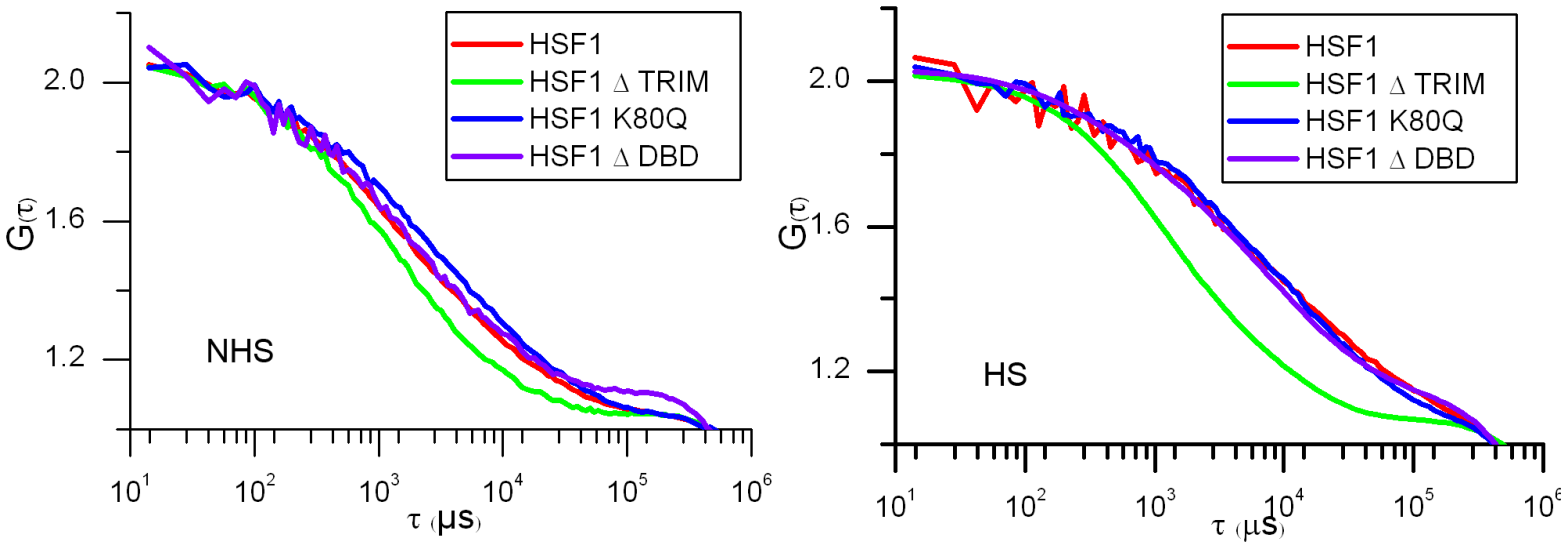
mFCS analysis of the WT HSF1 and mutants in unstressed and stressed cells. Comparison between the dynamics of WT HS1 and mutants analyzed by mFCS. Autocorrelation function (ACF) curves of HSF1 before (NHS) and after a one hour heat shock (HS). The ACF functions are normalized for molecule number. Abscissa is related to diffusion times. WT HSF1-eGFP (red), HSF1-ΔTRIM-eGFP (green), HSF1-K80Q-eGFP (blue) and HSF1-ΔDBD-eGFP (purple).

HeLa cell nuclei were analyzed outside nucleoli and nSBs (in the case of heat-shocked cells). The same cells were analyzed before and after one hour heat shock. Figure 3 shows the mean normalized autocorrelation function (ACF) curves before (NHS) and after heat shock (HS). The temporal behaviors of the ACF revealed differences in HSF1-eGFP dynamics. A slowing down in HSF1-eGFP dynamics was observed for all HSF1 constructs except for HSF1 depleted of its trimerization domain. Indeed, in that case, HSF1 dynamics is not significantly affected by a heat shock. In unstressed cells, HSF1 ΔTRIM-eGFP is even slightly more rapid than WT HSF1-eGFP (Figure 3).

**Figure 4:**
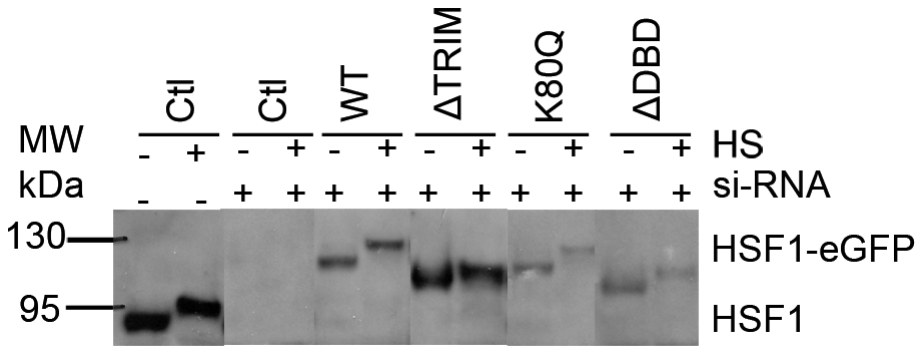
Expression levels of HSF1 and HSF1-eGFP in stable HeLa cell lines. HSF1 western blotting of full protein extracts before (−) and after one hour heat shock (+), before (−) or after (+) HSF1-siRNA treatment, in control cells (Ctl), in cell lines expressing the full length HSF1-eGFP (wt), the HSF1-eGFP mutant deleted of the trimerization domain (ΔTRIM), the HSF1-eGFP with the punctual mutation (K80Q) and the HSF1-eGFP mutant deleted for the DNA binding domain (ΔDBD). Only one band (>95 kDA) corresponding to –eGFP variant was observed after siRNA treatment, and the expression of endogenous HSF1 was efficiently reduced.

**Figure 5:**
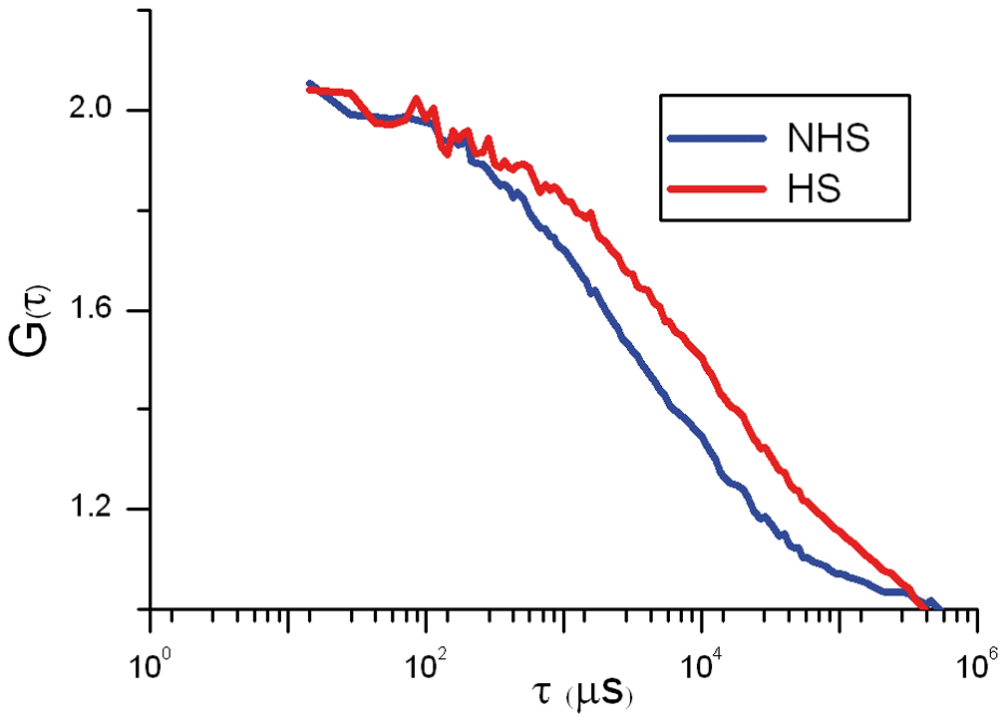
mFCS analysis of HSF1 -K80Q in cells knocked-down for endogenous HSF1. Dynamics of HSF1- K80Q-eGFP before (NHS, blue) and after a one hour heat shock (HS, red), in cells knocked-down for endogenous HSF1. mFCS acquisitions were made, 24 hours after two rounds of treatment with a siRNA against endogenous HSF1, followed by transfection of HSF1-eGFP- K80Q.

**Figure 6:**
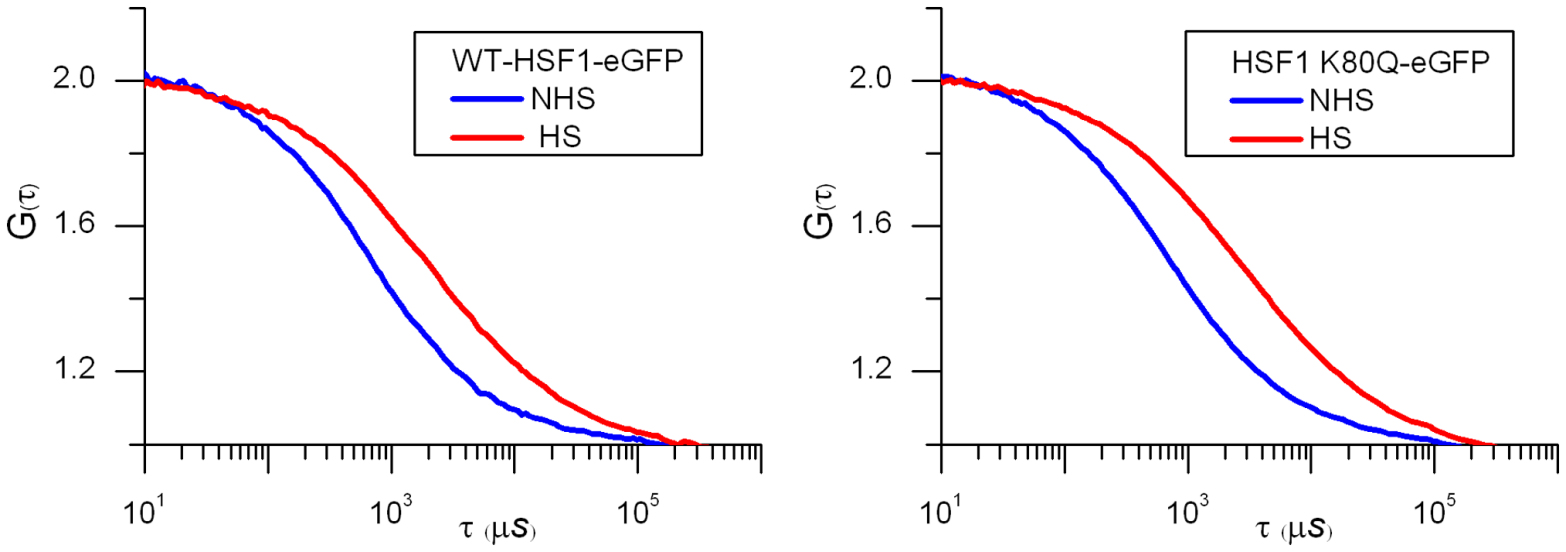
FCS analysis of HSF1 -K80Q in immortalized MEFs form HSF1 null mice. Dynamics of HSF1- K80Q-eGFP and WT HSF1-eGFP before (NHS, blue) and after 30 minutes heat shock (HS, red) in immortalized MEFs from HSF1 null mice.

**Figure 7:**
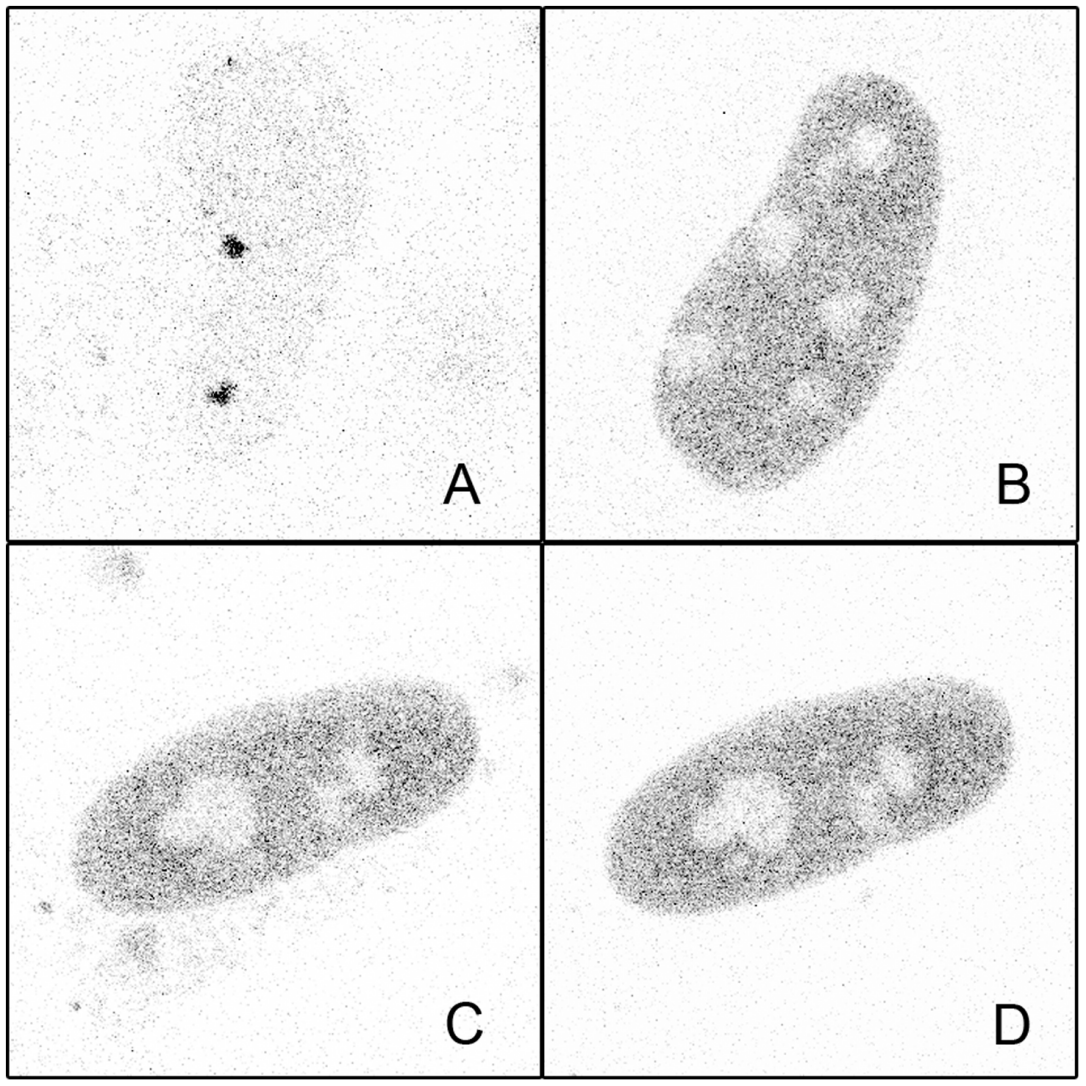
HSF1-K80Q prevents formation of nSBs and behaves as dominant negative. Confocal images of WT HSF1- tagRFP-T (A,C) and HSF1-K80Q-eGFP (B,D) after one hour heat shock in single transfected (A,B) and co-transfected (C,D) Hela cells. nSBs are only detected in single transfected WT HSF1-eGFP cells (A).

**Figure 8:**
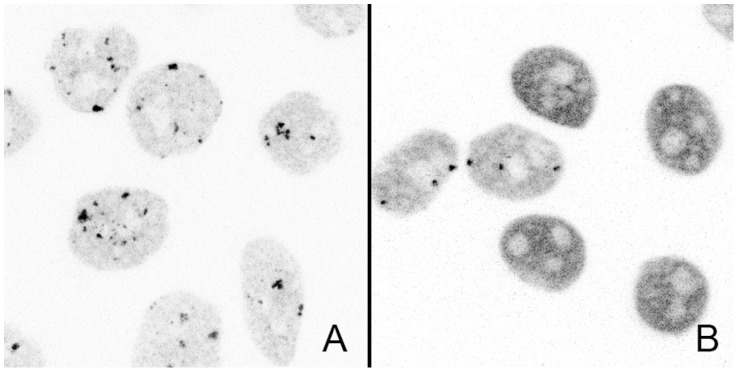
Intracellular distribution of endogenous HSF1 after heat shock. Confocal images of endogenous HSF1 after a one hour heat shock in stable WT and K80Q cell lines.

More surprising, while no targeting of ΔDBD or K80Q mutants to nSBs was observed upon stress, HSF1 dynamics was significantly slowed down following heat shock (Figure 3 blue and purple). Indirect DNA binding could not be excluded due to trimerization of HSF1-eGFP with endogenous HSF1. In order to clarify this point, endogenous HSF1 was knocked down using siRNAs. As shown on HSF1 western blot (Figure 4), the level of endogenous HSF1 was efficiently reduced. The cells were then transfected with a vector encoding a siRNA resistant HSF1-eGFP encoding mRNA. HSF1 K80Q-eGFP slow-down upon stress was still observed in the absence of endogenous HSF1 (Figure 5). The results were confirmed in immortalized HSF1 null MEF cells. As shown in figure 6, both WT and K80Q HSF1-eGFP were slowed down upon heat shock. Next, in transiently transfected cells, we checked that whatever the concentration of HSF1 K8OQ-eGFP we used, no nSBs were formed (Figure 7, A–B). In addition, co-transfection of WT HSF1-tagRFPT and HSF1 K80Q-eGFP showed that HSF1 K80Q-eGFP prevents the localization of WT HSF1-tagRFPT in nSBs (Figure 7 C–D). Finally, immuno-detection of endogenous HSF1 showed that only 40% of the cells displayed nSBs in the HSF1 K80Q-eGFP stable cell line (100% in WT HSF1-eGFP cell line). NSBs were also smaller and fewer in HSF1 K80Q-eGFP cells than in WT HSF1-eGFP cells (Figure 8). Our observations clearly show that WT and DNA binding mutants can trimerize with endogenous HSF1 and confirm a former work in which HSF1 K80Q was described as a dominant negative mutant, preventing the localization of WT HSF1 in nSBs [22]. In conclusion, although heterotrimerization occurs, these trimers do not display a capacity to form nSBs and do not bind to specific DNA binding sites.

### DNA Binding and Transactivating Capacities of WT HSF1 and HSF1 Mutants

**Figure 9:**
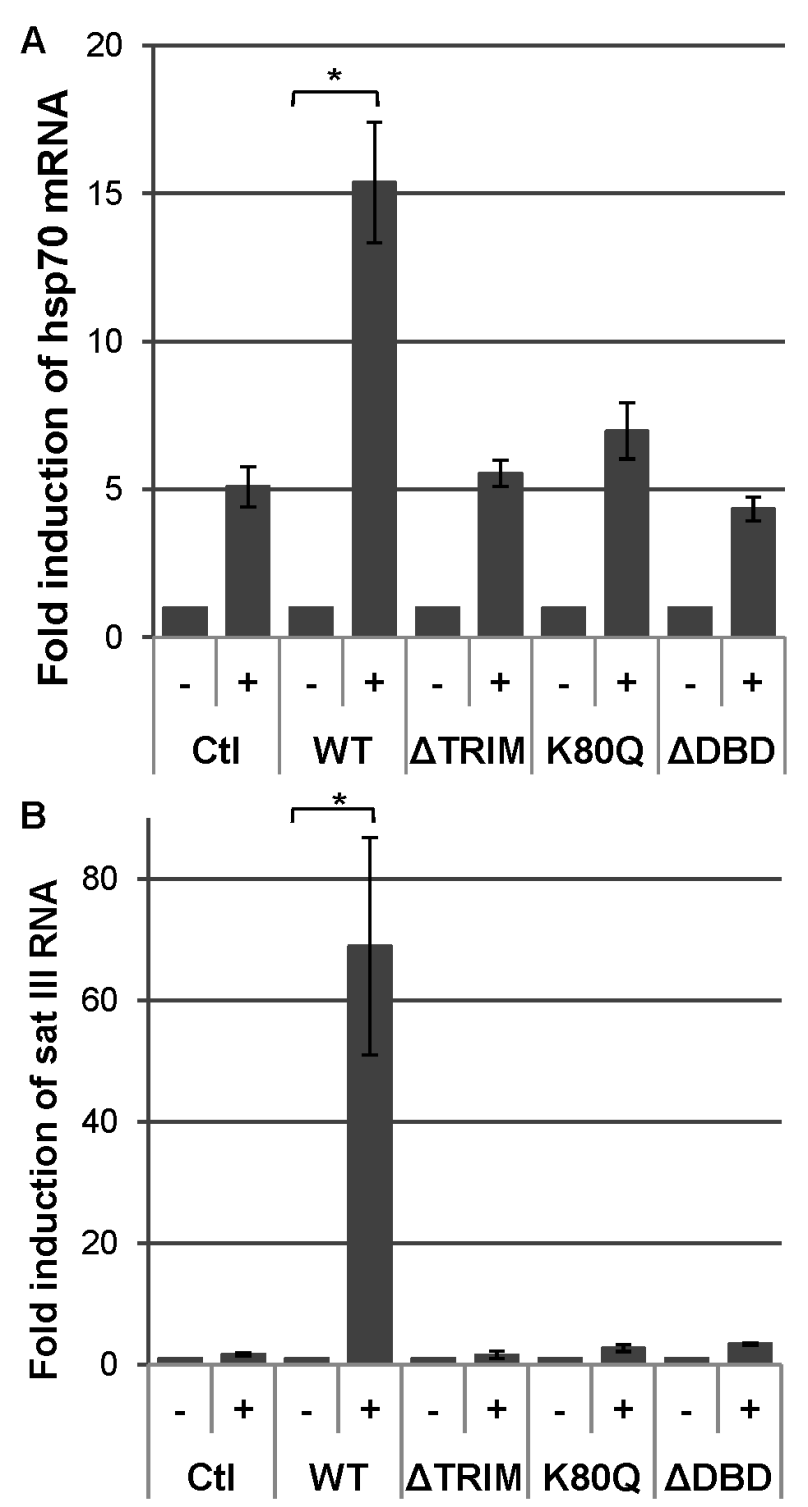
Hsp70 or sat III RNA expression following heat shock. Inductions of hsp70 (A) and sat III transcripts (B) were quantified by RT-QPCR before (-) and after a one hour HS followed by 4 hour recovery (+), in the absence of HSF1 (Ctl) or in the presence of the different HSF1 constructions. The results are expressed as fold induction in comparison to NHS condition, after normalization with GAPDH mRNA. The values correspond to the mean values obtained out of 6 (HSF1, HSF1-eGFP), 5 (HSF1-K80Q) or 3 (HSF1-ΔTRIM) experiments. Error bars correspond to S.E.M. * Significant difference (P<0.05).

Next, we sought to determine if these mutants were capable of interacting with chromatin in unstressed and stressed cells. First, we quantified the level of hsp70 gene and satellite III sequence expression by RT-QPCR, in the different cells expressing HSF1 and HSF1 mutants (Figure 9). The expression of the endogenous HSF1 gene was significantly reduced by siRNA (Figure 4).

A five-fold increase in the amount of hsp70 mRNA was observed in cells transfected with ΔDBD, K80Q and ΔTRIM mutants, similar to that of non-transfected cells (Ctl), revealing the persistence of a residual amount of endogenous HSF1, in HSF1 siRNA treated cells. No expression of sat III sequences was observed in these cells. A higher level of both hsp70 and sat III specific transcripts with regard to non-transfected cells was only observed in cells transfected with WT HSF1. Altogether, these data confirm the incapacity of HSF1 fusion proteins mutated in the DNA binding or trimerization domain of HSF1 to activate transcription.

**Figure 10:**
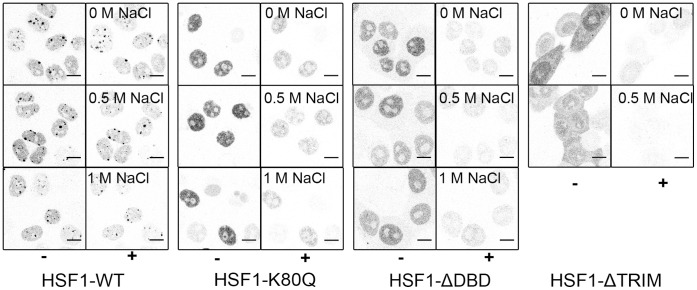
HSF1 DNA bound fraction after heat shock. CLSM images of HeLa cells expressing wild type and mutated HSF1 were displayed before (−) and after (+) 0.5% Triton-X100 permeabilization and increasing salt treatment (0.5 and 1 M NaCl). The same microscopic field is shown before and after treatment.

Second, although the DNA binding of HSF1-ΔTRIM, HSF1-K80Q and HSF1-ΔDBD to HSE or sat III repeats was clearly altered based on immunofluorescence images and RT-QPCR experiments, we sought to determine if these mutants were nonetheless capable of interacting with chromatin. To this end, we performed salt extraction on permeabilized cells transfected with eGFP fusion proteins. The level and distribution of fluorescence in permeabilized cells were analyzed and compared before and after treatment with different concentrations (absence, 0.5 M and 1 M) of salt (Table 1). In unstressed cells, WT HSF1, HSF1-−ΔTRIM, HSF1-K80Q and HSF1-ΔDBD were mostly solubilized as a result of cell permeabilization, even though a small residual fraction (20%), mainly observed with the WT-HSF1 mutant, remained bound to chromatin necessitating 0.5 M NaCl treatment to be solubilized (Table 1 “NHS”). In contrast, in stressed cells, only half of WT HSF1 was solubilized (Table 1 “HS”). Most HSF1 remained as a tightly bound fraction in interaction with specific target sequences as judged by the presence of fluorescence signal within nSBs in 1 M NaCl treated cells (Figure 10). In cells expressing HSF1-K80Q and HSF1-ΔDBD mutants, a significant amount of HSF1 was solubilized in response to detergent treatment (Table 1). However with these mutants, persistence of a fluorescence signal, even after 1 M NaCl treatment, revealed the existence of a small fraction (25%) of tightly bound HSF1 fraction displaying no specific DNA binding, as judged by the absence of nSBs (Figure 10). In contrast, HSF1− ΔTRIM mutant was mostly solubilized as a result of cell permeabilization, suggesting that this mutant does not significantly interact with chromatin upon stress.

**Table 1:** HSF1 Bound Fraction upon permeabilization(OM) and salt (0.5 M, 1 M).

	OM NaCl		0.5 M NaCl		1 M NaCl
	NHS	HS	NHS	HS	HS
**WT**	0.20±0.02	0.56±0.01	0.14±0.01	0.54±0.01	0.39±0.01
**ΔTRIM**	0.06±0.01	0.08±0.01	–	0.09±0.01	–
**K80Q**	0.08±0.01	0.41±0.01	0.07±0.01	0.32±0.01	0.25±0.01
**ΔDBD**	0.04±0.01	0.24±0.01	–	0.25±0.01	0.21±0.01
**eGFP**	–	0.01±0.01			

Comparison between data obtained for HSF1 (WT) and -HSF1 mutated in the trimerisation (ΔTRIM), DNA binding (ΔDBD) domain, punctual mutation in the DNA binding domain (K80Q) and for the protein eGFP alone.

**Figure 11:**
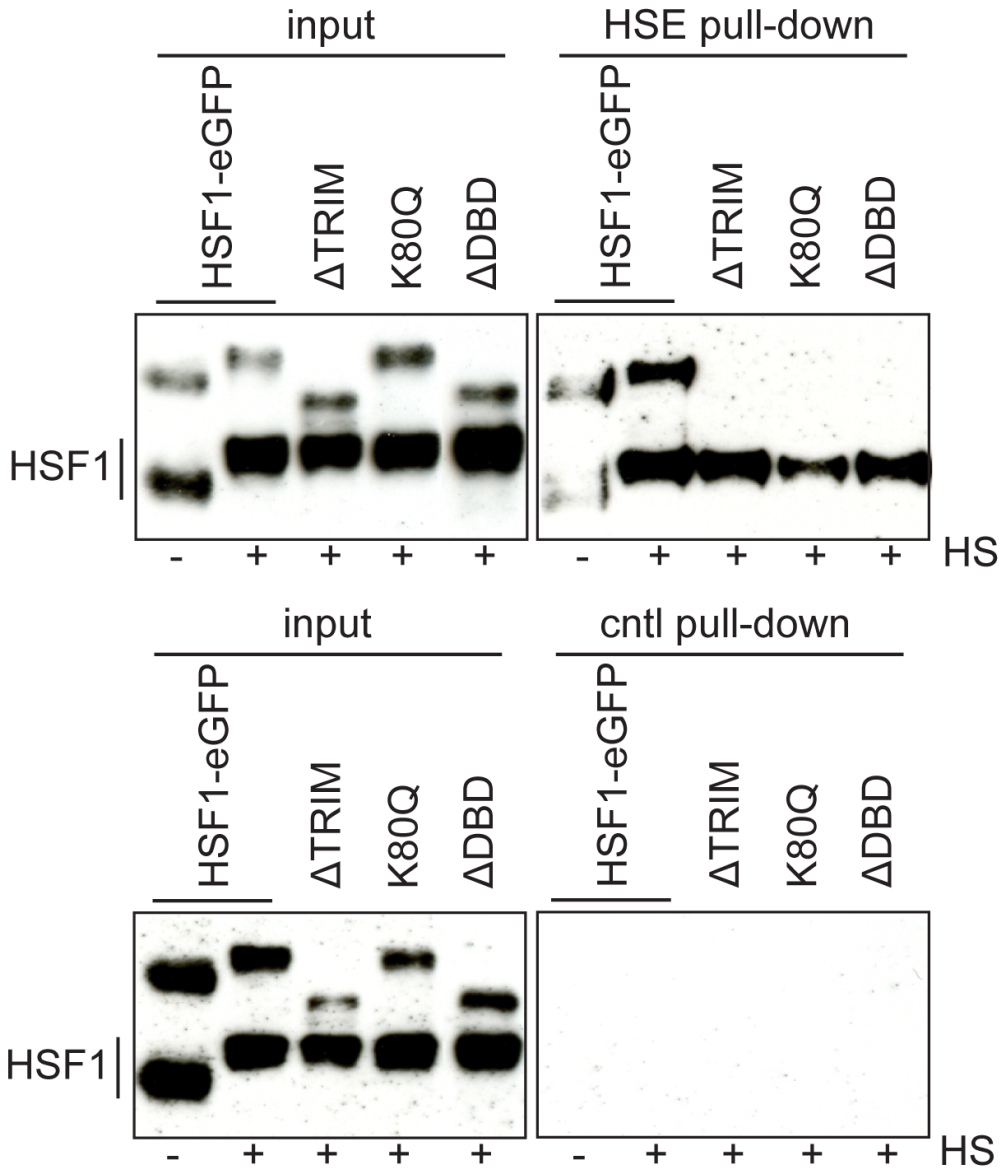
DNA binding of HSF1 to HSE after heat shock. Endogenous and full-length HSF1, but not HSF1 mutants, only bind to HSE. The biotinylated double-stranded oligonucleotides harboring the HSE motif were incubated with cellular extracts obtained from cell lines expressing the full length HSF1-eGFP (wt), the HSF1-eGFP mutant deleted of the trimerization domain (ΔTRIM), the HSF1-eGFP with the punctual mutation (K80Q) and the HSF1-eGFP mutant deleted of the DNA binding domain (ΔDBD). eGFP-variants were observed in all inputs (band >95 kDa). Only endogenous and full-length HSF1-eGFP bind to HSEs. Binding to HSE was neither observed for HSF1-eGFP mutants nor for WT HSF1 using control oligonucleotides.

Finally, direct binding of HSF1 to HSE was studied by means of a HSE oligonucleotide pull-down approach. In this experiment, we used biotinylated double-stranded oligonucleotides containing the consensus hsp70 gene HSE as HSF1 target. As shown in Figure 11, only endogenous and transfected full-length HSF1 were pulled down. Both trimerization and DNA binding domains are thus required for nSB formation and for HSF1 binding to HSE, upon heat shock, in agreement with already published results [22], [31].

In conclusion, wild-type HSF1 is tightly bound to chromatin in stressed cells and undergoes DNA-specific interactions. HSF1 mutations involving the DBD domains are still capable of forming interactions with chromatin, although in a sequence independent manner as judged by the inability of these mutants to form nSBs and to bind to HSEs.

### Estimation of HSF1 Molecular Complex Size

#### FCS modeling

According to our biochemistry experiments, a large fraction of HSF1 interacts with chromatin in stressed cells. Accordingly, ACF curves were fitted, assuming that HSF1 dynamics results from superimposed diffusion and DNA interactions. The diffusion constant (in µm^2^/s), the bound fraction and the dissociation rate (k_off_, s^−1^) were adjusted through a comparison with the experimental ACF data. The parameters (Table 2) confirm and complete the visual inspection of the ACF curves: in WT HSF1-eGFP, the diffusion constant (14 µm^2^/s in NHS, 10 µm^2^/s in HS) and the dissociation rate constant (k_off_, 45 s^−1^ in NHS, 25 s^−1^ in HS) decrease after heat shock, while the bound fraction (24% in NHS, 40% in HS) increases. Taking into account the eGFP diffusion constants measured in the nucleoplasm (37 µm^2^/s both in NHS and in HS cells [13]) and the different molecular masses of eGFP and HSF1-eGFP, the monomeric form of HSF1-eGFP (84 kDa) and the homo or hetero trimeric form (252 - 200 kDa) should display diffusion constants around 25 µm^2^/s and 18–19 µm^2^/s respectively. The significantly lower experimental values that we obtain suggest that HSF1 diffuses as a molecular complex, in unstressed and stressed cells. Therefore, we estimated the sizes of WT-HSF1 eGFP from the measured diffusion times and found 497 kDa and 1362 kDa in unstressed and stressed cells respectively.

**Table 2:** Diffusion and interaction parameters determined by mFCS.

	D (µm^2^s^−1^)		Bound (%)		k_off_ (s^−1^)	
	NHS	HS	NHS	HS	NHS	HS
**WT**	14	10	24	40	45	25
**ΔTRIM**	19	18	27	21	90	76
**ΔDBD**	18	8	27	34	81	34
**K80Q**	11	7	29	39	42	27
**K80Q, siRNA**	11	7	34	47	43	25
**eGFP**	37					

Comparison between data obtained for HSF1 (WT) and HSF1 mutated in the trimerisation (ΔTRIM), DNA binding (ΔDBD) domain, punctual mutation in the DNA binding domain in presence (K80Q) or absence of endogenous HSF1(K80Q, siRNA).

#### Fractionations on glycerol gradients

**Figure 12:**
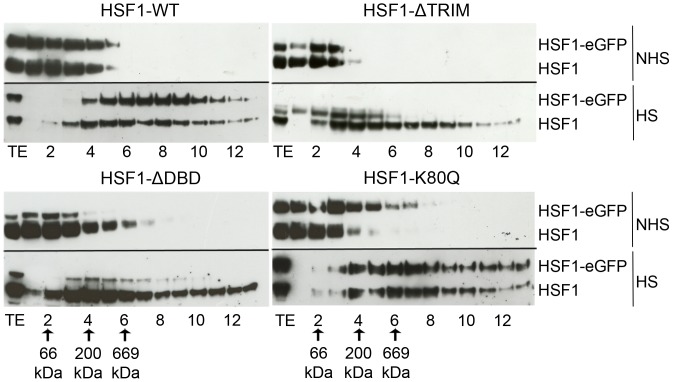
Sizes of HSF1 complex in unstressed and heat shocked HeLa cells using glycerol gradient fractionation. HSF1-eGFP and HSF1-K80Q-eGFP were estimated to 150 kD in NHS and superior to 669 kDa in HS. The complex size of HSF1- ΔTRIM-eGFP in NHS was estimated to 66–150 kDa in NHS and about 200 kDa in HS. The complex size of HSF1-ΔDBD-eGFP in NHS was estimated to 66–150 kDa in NHS and about 450 kDa in HS. TE: total extract before ultra-centrifugation. The fraction corresponding to standard proteins of masses 66, 200 and 669 kDa are indicated with arrows.

In order to confirm the presence of large HSF1-containing diffusing complexes in stressed cells, fractionation experiments using glycerol gradients were performed for all mutants. HSF1 trimerizes following heat shock and interacts with multiple chaperones, co-chaperones and other proteins during the different phases of its activation/recovery cycle. Fractionations on glycerol gradients were used to estimate the size of HSF1-eGFP molecular complexes from cellular extracts of unstressed and heat-shocked cells. As already published for WT HSF1 [28], very large HSF1 complexes (>669 kDa in HS vs 150 kDa in NHS) were present after heat shock (Figure 12). In addition, in unstressed and stressed cells, the general profile of HSF1 distribution was not significantly modified when the DNA binding capacity of HSF1 (K80Q and ΔDBD) was altered. A slight shift toward larger-size fractions was however observed, with the HSF1-ΔTRIM mutant, suggesting that, although this mutant does not form trimers in heat-shocked cells, it probably interacts with various partners in unstressed and stressed cells.

### Estimation of HSF1 Interaction

According to our salt extraction on permeabilized cells and FCS modeling, a large fraction (40%) of HSF1 interacts with chromatin in stressed cells. However, the dissociation rate constant estimated using FCS (k_off_, 25 s^−1^ in HS for WT HSF1-eGFP) is still high in the light of the percentage of HSF1 DNA interaction that we find to resist to a 1 M NaCl treatment. This could be due to the fact that continuous FCS acquisition underestimates long residence times because of the photobleaching of bound molecules [32]. So, FRAP analysis was performed in nuclei of unstressed and stressed cells, inside and outside nSBs.

**Figure 13:**
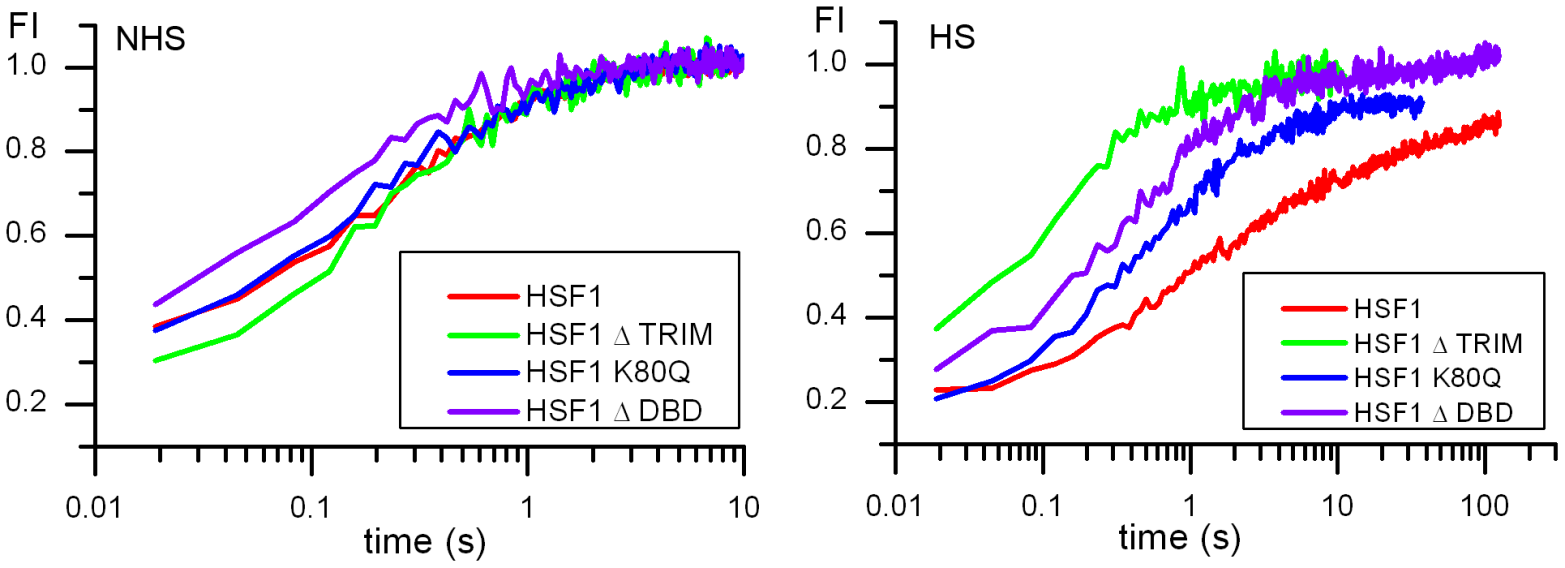
FRAP analysis of the WT HSF1 and mutants in unstressed and stressed cells. Fluorescence recovery curves after photobleaching of HSF1 full length-eGFP before (NHS) and after heat shock (HS) in the nucleus. WT HSF1-eGFP (red), HSF1-ΔTRIM-eGFP (green), HSF1-K80Q-eGFP (blue) and HSF1-ΔDBD-eGFP (purple).

As shown in Figure 13, whatever the HSF1 expressing construct considered, a full recovery was almost obtained both before and after heat shock, although with different kinetics. In agreement with the mFCS based approach, HSF1 dynamics was significantly slowed down following heat shock with both WT HSF1, ΔDBD and K80Q mutants, while the dynamics of the HSF1-ΔTRIM mutant was not significantly affected by a heat shock.

Moreover, significant differences in the dynamics of WT HSF1, ΔDBD and K80Q mutants were observed with the FRAP technique, when data were plotted on a logarithm scale (Figure 13). Upon stress, fluorescence recovery was faster with ΔDBD and to a lesser degree with K80Q when compared to WT HSF1-eGFP. As in FCS experiments, FRAP curves were fitted according to models taking into account diffusion, DNA binding capacity of HSF1 or both. In contrast, with FCS, a simple diffusion model is sufficient to fit FRAP data obtained in unstressed cells. Upon stress, the percentage of bound fractions found with ΔDBD or K80Q mutants were significantly smaller in comparison with WT HSF1 (14% for WT, 8% and ≈ 0% for K80Q and ΔDBD respectively) (Table 3).

**Table 3:** Diffusion and interaction parameters determined by FRAP.

	D (µm^2^s^−1^)		Bound (%)		k_off_ (s^−1^)	
	NHS	HS	NHS	HS	NHS	HS
**WT**	5.4	0.5		13.6		0.027
**ΔTRIM**	7.2	3.9				
**ΔDBD**	12.8	1.9				
**K80Q**	4.6	0.8		8.0		0.002
**K80Q, siRNA**	13.7	1.0		2.5		0.001
**eGFP**	35.6	42.1				

Comparison between data obtained for HSF1 (WT) and HSF1 mutated in the trimerisation (ΔTRIM), DNA binding (ΔDBD) domain, punctual mutation in the DNA binding domain in presence (K80Q) or absence of endogenous HSF1(K80Q, siRNA).

Diffusion constants measured by FRAP (after correction for diffusion during photobleaching [26]) are likely to represent an effective diffusion constant corresponding to both free diffusion and weak interactions of HSF1 molecules with chromatin, while interaction related parameters would correspond to tight interactions. The percentage of bound molecules and the dissociation rate constant are lower in FRAP than in FCS. Both parameters are likely to represent the bound fraction resistant to high concentration (1 M). They differ to those obtained by FCS, a technique allowing detection of weak interactions (>0.2 M) while under-estimating strong ones. Therefore, mFCS would favor the observation of diffusion and transient interactions occurring when transcription factors interact with chromatin through sliding or hopping, in search of specific DNA targets.

### Estimation of HSF1 Interactions within nSBs

**Figure 14:**
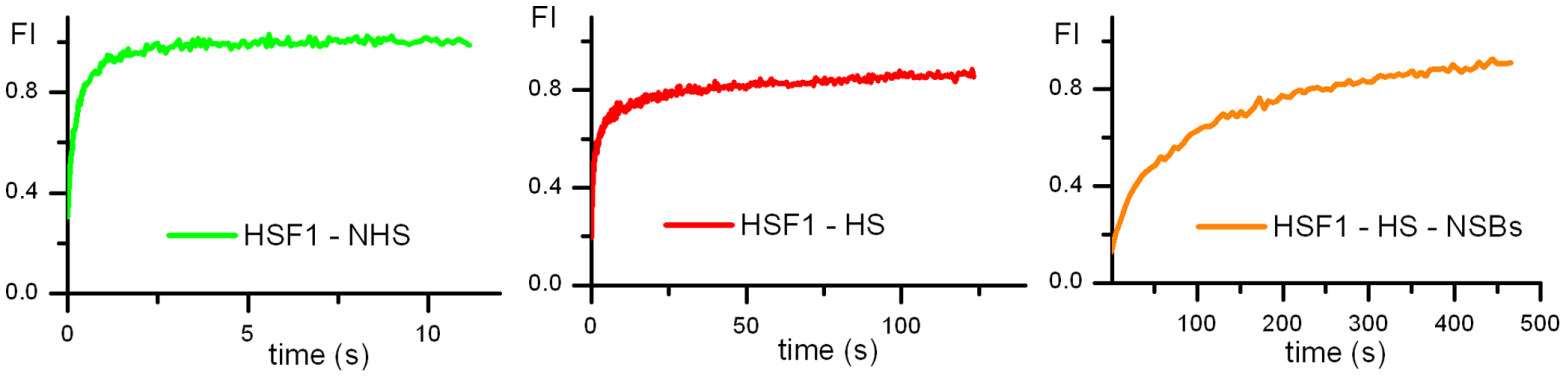
FRAP analysis of the WT HSF1 in and out nuclear stress bodies. Fluorescence recovery curves after photobleaching of HSF1 full length-eGFP before (NHS) and after heat shock (HS) in the nucleus outside nSBs (green and red) and in nSBs (orange).

In contrast to FCS, FRAP makes it possible to analyze the dynamics of HSF1 in nuclear stress bodies (nSBs), where specific HSF1 DNA targets are concentrated. Visual inspection of Figure 14 shows that the fluorescence recovery half time is much slower within nSBs than in the nucleoplasm. After a one hour heat shock, the half time increases from 0.2 s to 3 s in the nucleoplasm of unstressed and stressed cells respectively and to 65 s within nSBs (Figure 14). Full recovery times were obtained in nSBs in about 600 s. A detailed understanding and modeling of the kinetics of recovery within nSBs would necessitate to take into account the heterogeneous distribution of fluorescence between the bright nSBs and the dimmer surrounding area.

## Discussion

In this paper, FCS and FRAP were used as complementary approaches to analyze HSF1 dynamics in living cells. FCS is a more appropriate technique to determine the diffusion constant of the free mobile HSF1 fraction and therefore the size of diffusing complexes. In contrast, FRAP allows a better estimation of the percentage and dissociation rate constant of tightly bound HSF1 fraction to DNA. Indeed, based on the laser power and on the photobleaching yield of eGFP molecules, the minimum dissociation rate that can be measured by FCS is in the order of 3 s^−1^, in agreement with the minimum dissociation rate value that we found by analyzing the distribution of the k_off_ constants. Using FRAP, it is difficult to distinguish diffusion from weak interactions since the cross section area of the analyzed region is 80 fold greater in size than the one used in FCS analysis. As a consequence, in FRAP, weak interactions are incorporated in the measurement of the effective diffusion constant, making it smaller than the true diffusion one. Whatever the conditions considered (stressed or unstressed, outside or inside nSBs), the two techniques provide complementary assessments of dynamical processes involved in the heat shock response.

As expected, both FCS and FRAP techniques show that WT HSF1 dynamics is slowed down upon stress activation. This slowing down is not likely to be due to global stress-induced changes in nuclear organization since the diffusion constant of eGFP does not significantly vary upon heat-shock [13]. mFCS estimations suggest that HSF1 mainly diffuses as a complex. In unstressed cells, we found that the size of HSF1 complex(es) is close to 497 kDa, in agreement with the fact that HSF1 interacts with various chaperones and other proteins [14], [15], [33], [34], [35]. In stressed cells, our estimated size of the FCS complex(es) (1362 kDa) is larger than the molecular mass of HSF1-protein complex(es) found in the literature (700–800 kDa). The diffusion-reaction model that we use, allows a good discrimination between diffusion and interaction parameters. Although we cannot exclude that the size of the complex is slightly overestimated, fractionation experiments however confirm that complex(es) larger than 700 kDa do exist in living cells. In stressed cells, mFCS, FRAP and salt experiments show that WT HSF1 interactions with chromatin involve both direct DNA binding with HSE and sat III sequences, and a more transient sequence-independent chromatin binding likely corresponding at least in part, to HSF1 search for more specific targets.

Upon stress, significant differences were observed for all mutants, when data were compared to WT HSF1-eGFP. The most significant difference was observed when the trimerization domain was deleted, resulting in a stress-independent nucleo-cytoplasmic distribution of HSF1. Deletion of the trimerization domain did not impede stress-induced post-translational modifications nor any changes in the size of HSF1 complexes but these modifications were not accompanied by changes in HSF1 dynamics. This observation is in agreement with salt extraction based experiments indicating that the HSF1-ΔTRIM mutant is mainly present as a soluble factor. The strong difference between HSF1-ΔTRIM dynamics and that of other HSF1 mutants confirms that the trimerization domain represents an essential step in HSF1 activation.

In contrast, quite surprisingly, when the DNA-binding capacity of HSF1 was impaired through punctual mutation or deletion of the whole DBD domain, HSF1 dynamics was still significantly slowed down, upon stress, although to a lesser extent than WT HSF1 as assessed by FRAP analysis. Similar to WT HSF1, these mutants diffuse as large complexes and interact with chromatin.

The slow-down that we observe with HSF1-ΔDBD and K80Q mutants is in agreement with our fractionation experiment showing that these mutants exist as large complexes, and with the 500 mM salt treatment showing that a fraction of them remains bound to chromatin. However, in vitro and in vivo experiments indicate that both mutants do not bind to HSE and sat III sequences, and their interactions with chromatin are transcriptionaly inefficient.

Since the dynamics of HSF1 K80Q does not significantly vary when HSF1 is knocked-down (siRNA treated cells) or absent (MEF cells derived from HSF1 null mice), the chromatin-bound fraction that we detect cannot be imputed to hetero-trimerization of HSF1 K80Q with endogenous WT HSF1. Moreover, we confirm a former work showing that HSF1 K80Q is unable to form nSBs either as homo or hetero-trimers and behaves as a dominant negative mutant, preventing the accumulation of WT HSF1 within nSBs [22]. Our data therefore suggest that the three DNA binding domains are needed for the recruitment of WT HSF1 to nSBs. A similar impact was also reported in the case of HSF2 deleted in its DNA binding domain. This mutant was also shown to impair the stress-induced redistribution of both HSF1 and HSF2 to nSBs [36].

We propose that the chromatin-bound fraction that we observed with the DBD mutants could reveal HSF1 continuous search for binding sites or other processes involving its association with partners. Our findings are in agreement with data obtained on p53 showing that p53 locates specific DNA targets by first binding at sequence-independent sites [2], [37].

Our data also suggest that HSF1 is more dynamic in HeLa cells than it is in Drosophila salivary gland [12]. Indeed, data reported by Yao et al [12] are significantly different, with longer half recovery time (15 s in NHS, >6 min in HS) using FRAP, and no slowing down following heat shock in the nucleoplasm using FCS. Difference of positioning in NHS cells (HSF specific target on polytenic chromosomes, nucleoplasm in HeLa cells), could explain these differences. However, in stressed cells, nSBs correspond to active transcription sites of satellite III sequences and can thus be compared to active hsp70 transcription sites in Drosophila. In stressed Drosophila cells, the FRAP curve reaches a plateau 6 min after photobleaching with a 0.6 partial recovery. The difference with our data could be due in part to differences in FRAP setup and standardization. In our FRAP experiments, cell mobility was taken into account in 3D acquisitions, so that a follow-up of the bleached nSBs could be done during the recovery process. In addition, a correction of photobleaching was performed, using the second nSB as a reference. The main advantage of this process is to compensate fluorescence loss during photobleaching and acquisition steps. Discrepancies in dynamics data could also result from the existence of cell specificity behavior. In that regard, Schaaf and Cidlowski [38] have reported a smaller FRAP half-recovery for the glucocorticoid receptor (GR) in HeLa cells than in COS-1 or HEK93 cell lines. These differences could result from differences in molecular crowding or in the number of DNA targets.

However, the half recovery time that we obtain within nSBs (65 s) is slower than that reported for nuclear receptors obtained using models of gene arrays. Most half recovery times obtained for activated nuclear receptors indicate more transient associations with promoters (t_1/2_<10 s ) [3], [4]. We must keep in mind that the half recovery time measured in nSBs as in the case of other gene arrays, could be over estimated. Indeed, a high concentration of DNA target sites is likely to favor the reassociation of dissociated bleached proteins to new free DNA targets.

In conclusion our work shades new light on the dynamics of HSF1 in living cells and reveals the existence of low affinity interactions of HSF1 independent of the presence of its DNA binding domain. It also illustrates the importance of combining different approaches for the determination of diffusion and interaction parameters. Our work also reinforces the idea that these parameters might not only depend on the conditions of acquisition but also on the biological model considered.
